# Assessing the Effects of Parthenolide on Inflammation, Bone Loss, and Glial Cells within a Collagen Antibody-Induced Arthritis Mouse Model

**DOI:** 10.1155/2020/6245798

**Published:** 2020-03-04

**Authors:** B. Williams, F. Lees, H. Tsangari, M. R. Hutchinson, E. Perilli, T. N. Crotti

**Affiliations:** ^1^Adelaide Medical School, University of Adelaide, Adelaide SA, Australia 5005; ^2^ARC Centre for Excellence for Nanoscale Biophotonics, University of Adelaide, Adelaide SA, Australia 5005; ^3^Medical Device Research Institute, College of Science and Engineering, Flinders University, Tonsley SA, Australia 5042

## Abstract

Rheumatoid arthritis is characterised by a chronic inflammatory response resulting in destruction of the joint and significant pain. Although a range of treatments are available to control disease activity in RA, bone destruction and joint pain exist despite suppression of inflammation. This study is aimed at assessing the effects of parthenolide (PAR) on paw inflammation, bone destruction, and pain-like behaviour in a mild collagen antibody-induced arthritis (CAIA) mouse model. CAIA was induced in BALB/c mice and treated daily with 1 mg/kg or 4 mg/kg PAR. Clinical paw inflammation was scored daily, and mechanical hypersensitivity was assessed on alternate days. At end point, bone volume and swelling in the paws were assessed using micro-CT. Paw tissue sections were assessed for inflammation and pre-/osteoclast-like cells. The lumbar spinal cord and the periaqueductal grey (PAG) and rostral ventromedulla (RVM) regions of the brain were stained for glial fibrillary acidic protein (GFAP) and ionised calcium-binding adaptor molecule 1 (IBA1) to assess for glial reactivity. Paw scores increased in CAIA mice from days 5-10 and were reduced with 1 mg/kg and 4 mg/kg PAR on days 8-10. Osteoclast-like cells on the bone surface of the radiocarpal joint and within the soft tissue of the hind paw were significantly lower following PAR treatment (*p* < 0.005). GFAP- and IBA1-positive cells in the PAG and RVM were significantly lower following treatment with 1 mg/kg (*p* < 0.0001 and *p* = 0.0004, respectively) and 4 mg/kg PAR (*p* < 0.0001 and *p* = 0.001, respectively). In the lumbar spinal cord, IBA1-positive cells were significantly lower in CAIA mice treated with 4 mg/kg PAR (*p* = 0.001). The findings indicate a suppressive effect of both low- and moderate-dose PAR on paw inflammation, osteoclast presence, and glial cell reactivity in a mild CAIA mouse model.

## 1. Background

Rheumatoid arthritis (RA) is a chronic systemic disorder characterised by joint inflammation, synovial hyperplasia, and associated destruction of the cartilage and bone. Pain is associated with this joint destruction and is one of the most debilitating symptoms reported by RA patients [1, 2]. The pathogenesis of RA involves chronic infiltration of immune cells into the synovial joints and production of proinflammatory cytokines including tumour necrosis factor alpha (TNF-*α*), interleukin-1 beta (IL-1*β*), and interleukin-6 (IL-6) [3]. These cytokines not only prolong the inflammatory response within the synovial joints, but their overproduction promotes bone destruction [4, 5].

The inflammatory cytokines mentioned above have been shown to sensitise peripheral nerves, resulting in increased pain sensitivity [6]. Further to this, glial cells have been identified as being key drivers behind central sensitisation and hypersensitivity in chronic inflammatory states [7]. The development of mechanical hypersensitivity has previously been reported in mice with inflammatory arthritis, with an increased glial cell number also observed [8]. However, mechanical hypersensitivity was observed prior to signs of inflammation, suggesting that the timing of inflammation and pain in RA may not necessarily coincide [8]. Exaggerated pain, known as hypernociception can persist in RA even in the absence of clinical symptoms [9]. The disconnect between the presence of pain in the absence of peripheral inflammation, as observed in RA, supports the new neuroimmune body of evidence (as reviewed by Grace et al. [10]).

Currently, disease-modifying antirheumatic drugs (DMARDs) target inflammation, allowing chronic bone erosion to progress. Direct targeting of osteoclast differentiation has been shown to slow the progression of bone erosion in RA patients [11]; however, inflammation of the synovial joints ensued. To date, there are limited studies which have assessed the effect of anti-inflammatory treatments on inflammation, joint destruction, and glial reactivity in RA pathogenesis, simultaneously.

Parthenolide (PAR) is a sesquiterpene lactone found in the Asterceae family of medicinal plants, including feverfew [12], and is reported to have anticancer as well as anti-inflammatory, antibone resorptive, and antinociceptive actions in both *in vitro* and *in vivo* models [13–16]. *In vitro*, PAR has been shown to prevent osteoclast formation and bone resorption [17], as well as inhibit the effects of IL-1*β* and TNF-*α* on human chondrocytes [18], which are key driving factors of RA pathogenesis. *In vivo* studies have shown that PAR (0.5 and 1 mg/kg) blocks LPS-induced osteolysis [17] and inhibits wear particle-induced surface bone loss in murine calvarial models [19]. Within a collagen-induced arthritis (CIA) rat model, PAR 1 mg/kg reduced inflammation and pannus formation [18]. Of note, no clear reduction in bone erosion was found and mechanical hypersensitivity was not investigated [18]. In a clinical trial in migraine sufferers, PAR was found to have no major safety concerns [20], although further studies are warranted.

To our knowledge, current studies have not yet investigated the effect that PAR has, directly or indirectly, on the central nervous system and pain, within a collagen antibody-induced arthritis (CAIA) mouse model. Therefore, the current study was aimed at investigating whether PAR (low and moderate dose) would reduce inflammation, bone loss, mechanical hypersensitivity, and glial reactivity in a mild CAIA mouse model.

## 2. Methods

This study was approved by the Animal Ethics Committee of the University of Adelaide (M-2015-255) and complied with the National Health and Research Council (Australia) Code of Practice for Animal Care in Research and Training (2014). Mice were housed in approved conditions on a 12-hour light-dark cycle. Food and water were provided ad libitum and mice were provided with waterproof soft rubber matting as bedding prior to disease induction.

### 2.1. Collagen Antibody-Induced Arthritis Model

Thirty-two female BALB/c mice aged six to eight weeks were obtained from the University of Adelaide Laboratory Animal Services and randomly allocated to control (no arthritis or treatment), CAIA (arthritis with no treatment), CAIA+PAR 1 mg/kg (arthritis treated with 1 mg/kg PAR), and CAIA+PAR 4 mg/kg (arthritis treated with 4 mg/kg PAR).

Arthritis was induced by an intravenous injection, via the tail vein, with 150 *μ*l (1.5 mg/mouse) of a cocktail of anti-type II collagen monoclonal antibodies (Arthrogen-CIA Arthritogenic Monoclonal Antibodies, Chondrex Inc., Redwood, WA, USA), followed by an intraperitoneal injection of 20 *μ*l (10 *μ*g/mouse) of *E. coli* lipopolysaccharide (LPS) on day 3, as previously described [21–23]. Control animals were injected with 200 *μ*l phosphate-buffered saline (PBS) at both time points.

PAR (Enzo Life Sciences, Inc., Sapphire Bioscience, NSW, Australia) was administered at 1 mg/kg in 200 *μ*l of 10% DMSO in PBS [18], or at 4 mg/kg in 200 *μ*l of 0.8% DMSO in PBS via intraperitoneal injection, daily on alternating sides, from day 4 to day 10. Mice were monitored daily for body weight and factors of general health using an approved clinical record sheet for arthritis studies. Clinical paw swelling was examined daily for the presence of redness, tenderness, swelling, and inflammation in all paws, by two blinded observers, using a previously described clinical paw scoring method [22, 24].

### 2.2. Assessment of Mechanical Hypersensitivity

Mechanical hypersensitivity was assessed in the hind paws using the von Frey paw withdrawal test on alternate days (i.e., days 2, 4, 6, 8, and 10) as BALB/c mice develop a tolerance to the behavioural test [8, 25]. Mice were placed in plastic cages with a wire mesh bottom to allow full access to the hind paws, and behavioural accommodation was allowed for 15 minutes. Behavioural testing followed the previously published Dixon up-down method, and the 50% paw withdrawal threshold was calculated [25]. The hind paw with the lowest 50% probability of paw withdrawal was identified for examination of mechanical hypersensitivity as the severity of inflammation between paws can differ following CAIA induction [22, 23].

### 2.3. Microcomputed Tomography Analysis

On day 11, transcardial perfusions were performed under anaesthetic (175 mg/kg sodium pentobarbital) using 4% paraformaldehyde. Brain, spinal cord, and paw tissues were then collected and underwent postfixation for 48 hours for micro-CT and histopathological assessment.

Bone volume (BV) and paw volume (PV, indicator of soft tissue swelling) of the front and hind paws were assessed using a microcomputed tomography (micro-CT) scanner (SkyScan 1076, Bruker, Kontich, Belgium) [22, 26]. The scan settings were as follows: X-ray source voltage 55 kV, current 180 *μ*A, isotropic pixel size 8.5 *μ*m, 0.5 mm thick aluminium filter, 0.6 rotation step, frame averaging of 1, and scan time of 35 minutes. Cross-sectional images of all paws were reconstructed (N-Recon software, Bruker, Kontich, Belgium), saved in 8-bit format and realigned with the long axis of each paw along the inferior-superior direction of the images (Dataviewer, Bruker, Kontich, Belgium), as previously described [26].

For front paws, a cylindrical volume of interest (VOI; 4.5 mm diameter, 2.4 mm length) was used over 280 contiguous cross sections, starting 200 cross sections (1.7 mm) distally to the growth plate of the radiocarpal joint and extending to 80 cross sections (0.68 mm) proximally, for analysis of bone and soft tissues [22]. In the hind paws, 600 cross sections (5.1 mm length), extending from the posterior surface of the calcaneus through the proximal tarsal and metatarsal bones, were used for BV analysis, along with a polygonal VOI traced around the calcaneus, tarsal and metatarsal bones, excluding the tibia and fibula. PV analysis used a cylindrical VOI, with 200 cross sections (5.5 mm diameter, 1.7 mm length), extending from the most posterior aspect of the metatarsal bones, excluding the calcaneus and including the cuboid. For these VOIs, BV (mm^3^) and PV (mm^3^) were quantified in 3D using uniform thresholding (CT Analyser software, V1.15.40, Bruker) [22, 26].

### 2.4. Histological Analysis of the Radiocarpal Joints and Hind Paws

Front and hind paws were decalcified using 10% ethylene diaminetetraacetic acid (EDTA), processed into paraffin and sagittal sections cut (5 *μ*m) for routine haematoxylin and eosin (H&E) and tartrate resistant-acid phosphatase (TRAP) staining. Histological evaluation of H&E sections for the presence of inflammatory cells, cartilage and bone degradation, and pannus formation was carried out using a previously published semiquantitative scoring method [27, 28].

The number of multinucleated TRAP-positive cells (>3 nuclei) was counted in a consistent region of interest (2.16 mm^2^), to include cells found on the bone surface [27] and within the surrounding soft tissue of the r joints and hind paws [22].

### 2.5. Histological Analysis of Spinal Cord and Brain Tissue

Immunohistochemical detection of glial fibrillary acidic protein (GFAP) and ionised calcium-binding adaptor molecular 1 (IBA1) was conducted on serial coronal sections (5 *μ*m) of the lumbar spinal cord (L3-L5) and brain. Sections were dewaxed and dehydrated in 100% ethanol, before endogenous peroxidase activity was removed by 0.5% hydrogen peroxidase. Slides were washed in 1x PBS (pH 7.4, 2 × 5 minutes) for GFAP and 1x PBS+0.3% Triton X (BOH Chemicals, Australia, 2 *x* 5 minutes) for IBA1. Following heat-mediated antigen retrieval in citrate (0.1 mol/l, pH 6.0), nonspecific binding was blocked by 3% normal horse serum (NHS; Sigma-Aldrich) for 30 minutes. Primary antibodies (GFAP; Dako 72.5 *μ*g/ml, catalogue #Z0334 and IBA1; Wako 0.05 *μ*g/*μ*l, catalogue #019-19741) diluted in 3% NHS were incubated overnight at room temperature, followed by incubation with a secondary goat biotinylated anti-rabbit IgG antibody (6 *μ*g/ml; Vector Laboratories) for 30 minutes. Following 1x PBS/1x PBS+0.3% Triton X wash, slides were incubated with streptavidin peroxidase conjugate (2 *μ*g/ml; ThermoFisher Scientific) and developed with diaminobenzidine and counterstained with haematoxylin [29].

IBA1-positive cells were counted within the lumbar spinal cord (0.38 mm^2^ region of interest) by a blinded observer. Positive cells were also counted in the PAG (2.72 mm^2^ region of interest) and RVM (0.079 mm^2^ region of interest) regions in the brain, as these regions have been demonstrated to contribute to nociceptive processing [30, 31]. GFAP-positive cells were counted in the same regions of the lumbar spinal cord and brain using the software Fiji and colour deconvolution method [32] and a threshold of 125 ± 5 pixels^2^ and 100 ± 5 pixels^2^, respectively.

### 2.6. Statistical Analysis

Statistical analysis utilised GraphPad Prism® software (V7.03; GraphPad Software, La Jolla, CA, USA) and SPSS Statistics (V25; IBM SPSS Software, NSW, Australia). Paw inflammation was assessed using two-way ANOVA. For von Frey analysis, a linear mixed-effects model was performed and for repeated measurement over time using a variance component covariance structure. Assumptions of a linear model were found to be upheld by inspection of histograms and scatter plots of residuals and predicted values. An interaction of group (1 = control, 2 = CAIA, 3 = CAIA+PAR 1 mg/kg, and 4 = CAIA+PAR 4 mg/kg) and day (day of test) was included. For micro-CT and paw and brain histology data, differences among groups were analysed using the nonparametric Kruskal-Wallis test and if significant, differences between two groups were analysed using the Mann-Whitney *U* test. Area under the curve (AUC) analysis was conducted on lumbar spinal cord cell counts. Statistical significance was assessed post hoc using *t*-tests between groups. All values shown are mean ± standard error of the mean (SEM), and the significance level was set to *p* < 0.05.

## 3. Results

### 3.1. Assessment of Local Paw Inflammation and Mechanical Hypersensitivity

**Figure 1:**
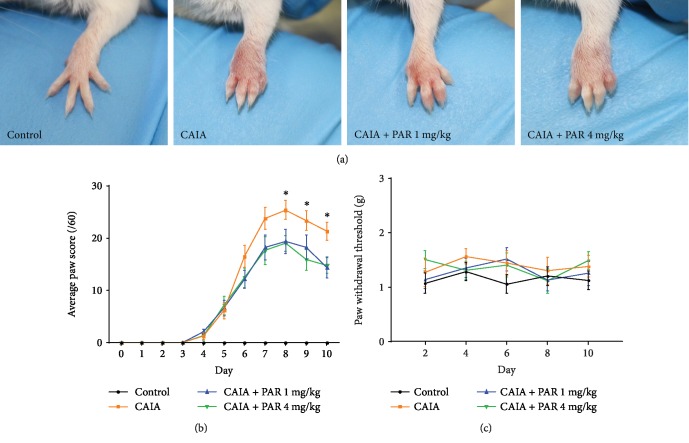
Evaluation of local inflammation and mechanical hypersensitivity of mouse paws: (a) representative macroscopic appearance of the front paws at day 7 postarthritis induction; (b) average paw scores of each group over the 10-day model. Control mice had a paw score of 0 at each time point; (c) mean tactile paw withdrawal threshold of each group on alternate days throughout the 10-day model. Error bars represent SEM (*n* = 8 mice per control, CAIA+PAR 1 mg/kg, and CAIA+PAR 4 mg/kg; *n* = 6 mice per CAIA; ^∗^*p* < 0.05).

Induction of CAIA resulted in significant redness, tenderness, and inflammation in all paws of disease groups following LPS administration on day 3 (Figure 1(a)). CAIA mice exhibited significantly greater paw scores compared to control mice from day 5 to day 10 (*p* < 0.0003; Figure 1(b)). On days 8, 9, and 10, PAR 4 mg/kg-treated mice had significantly lower paw scores compared to CAIA mice (*p* = 0.041, *p* = 0.019, and *p* = 0.017, respectively; Figure 1(b)). On day 10, PAR 1 mg/kg-treated mice also had significantly lower paw scores compared to CAIA mice (*p* = 0.032; Figure 1(b)). There was no significant difference in paw withdrawal thresholds between control and CAIA mice, as well as between CAIA mice and PAR 1 mg/kg and 4 mg/kg- treated mice (Figure 1(c)).

### 3.2. Micro-CT Analysis of Bone Volume (BV) and Paw Volume (PV)

**Figure 2:**
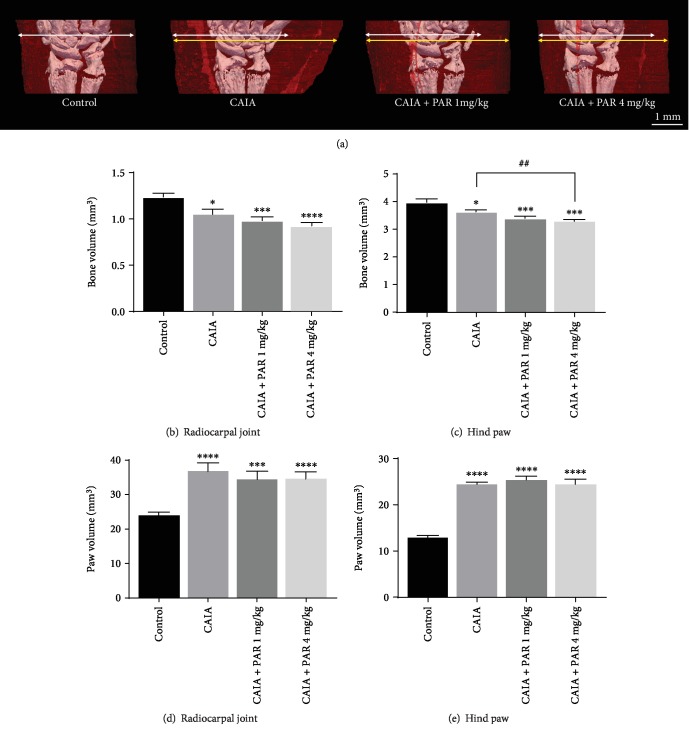
Assessment of bone volume (BV) and paw volume (PV) in mouse paws by high-resolution micro-CT: (a) three-dimensional micro-CT models of the radiocarpal joint and surrounding soft tissue (indicated in red) in the right paw. White arrows represent soft tissue volume for control. Yellow arrows represent the soft tissue volume for each disease group and highlight the difference in soft tissue and volume observed. Mean BV in the radiocarpal joint and hind paw (b and c, respectively) and mean PV in the radiocarpal joint and hind paw (d and e, respectively) expressed in mm^3^, as assessed by micro-CT analysis at day 11. Error bars represent SEM (*n* = 16 paws per control, CAIA+PAR 1 mg/kg, and CAIA+PAR 4 mg/kg; *n* = 12 paws per CAIA; ^∗^*p* < 0.05, ^∗∗∗^*p* = 0.0001, ^∗∗∗∗^*p* < 0.0001 compared to control; ^##^*p* = 0.04 compared to CAIA).

BV measured in the radiocarpal joints was significantly lower in CAIA mice (1.05 ± 0.05 mm^3^) compared to control mice (1.24 ± 0.05 mm^3^; *p* = 0.017; Figures 2(a) and 2(b)). PAR 1 mg/kg- (0.98 ± 0.04 mm^3^) and 4 mg/kg- (0.93 ± 0.04 mm^3^) treated mice tended to have slightly lower BV in the radiocarpal joint compared to CAIA mice, however, this was not statistically significant.

**Figure 3:**
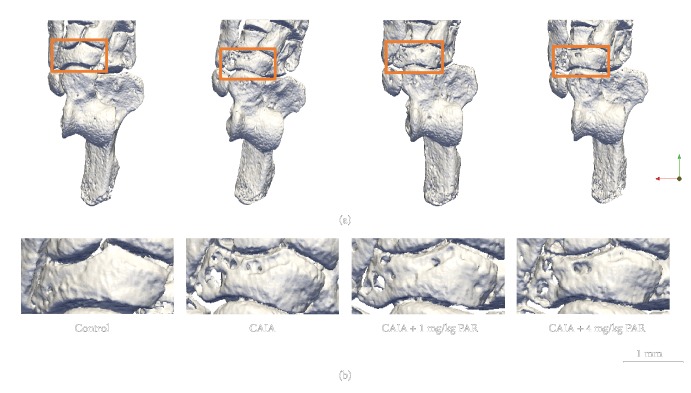
Representative three-dimensional micro-CT models of the hind paw showing pitting in the navicular: (a) superior view of left hind paws from the control (first column), CAIA (second column), CAIA+PAR 1 mg/kg (third column), and CAIA+PAR 4 mg/kg groups (fourth column). Orange boxes identify the navicular which is presented at greater magnification (b) to emphasise the pitting present in this bone.

BV in the hind paws was significantly lower in CAIA mice (3.64 ± 0.08 mm^3^; *p* = 0.01) and PAR 1 mg/kg- (3.40 ± 0.07 mm^3^; *p* = 0.0004) and 4 mg/kg- (3.30 ± 0.07 mm^3^; *p* = 0.0001) treated mice, compared to control mice (3.98 ± 0.14 mm^3^; Figure 2(c)). PAR 4 mg/kg-treated mice had significantly lower BV compared to CAIA mice in the hind paw (*p* = 0.004; Figure 2(c)). There was evidence of pitting visually observed on the 3D micro-CT images of the navicular bone of the hind paws (Figure 3) of CAIA groups both with and without PAR treatment.

CAIA mice (37.14 ± 2.07 mm^3^) had a significantly higher PV in the radiocarpal joint compared to control mice (24.17 ± 0.82 mm^3^, *p* < 0.0001; Figure 2(d)). PAR 1 mg/kg- and 4 mg/kg- treated mice showed lower PV measurements in the radiocarpal joint (34.74 ± 2.05 mm^3^ and 34.94 ± 1.75 mm^3^, respectively) compared to CAIA mice. However, this was not significantly different (Figure 2(d)). There was no difference in PV between PAR treatment groups.

A significant increase in PV was also observed in the hind paws of CAIA mice (24.70 ± 0.53 mm^3^) and PAR 1 mg/kg- (25.61 ± 0.57 mm^3^) and 4 mg/kg- (24.65 ± 0.93 mm^3^) treated mice, compared to control mice (13.15 ± 0.33 mm^3^; *p* < 0.0001; Figure 2(e)). There was no significant difference in PV measured in the hind paws between CAIA and PAR treatment groups nor between the PAR 1 mg/kg and 4 mg/kg treatment groups.

### 3.3. Histological Analysis of the Radiocarpal Joint and Hind Paws

**Figure 4:**
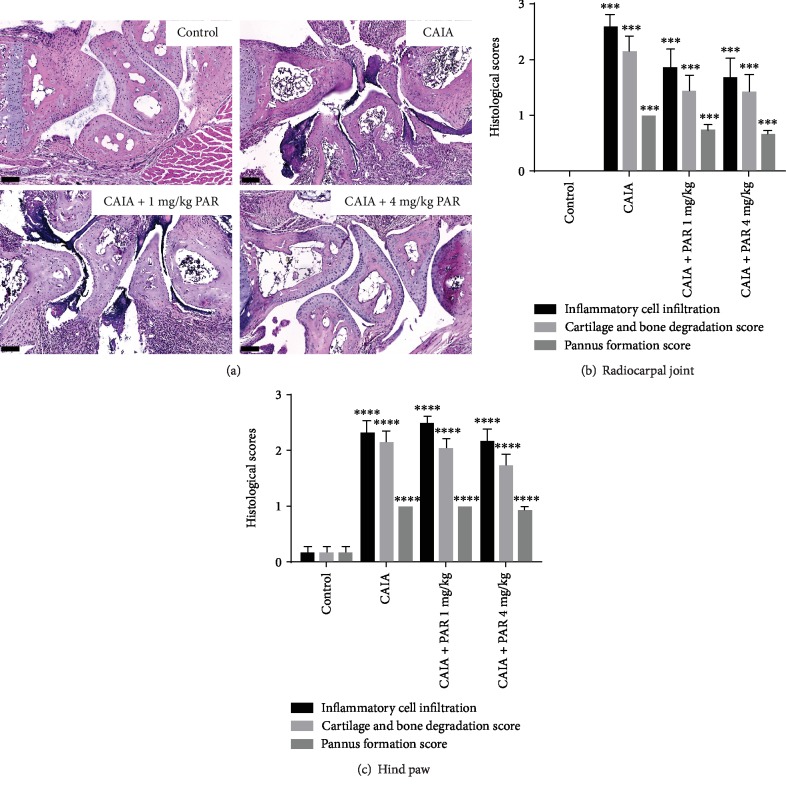
Histological assessment of the radiocarpal joint and hind paws: (a) representative haematoxylin and eosin- (H&E-) stained images of the radiocarpal joint (20x magnification; scale bars represent 100 *μ*m). Semiquantitative analysis of inflammatory cell infiltration, cartilage and bone degradation, and pannus formation in H&E-stained sagittal sections of the radiocarpal (b) and hind paws (c). Error bars represent SEM (*n* = 16 paws per control, CAIA+PAR 1 mg/kg, and CAIA+PAR 4 mg/kg, *n* = 12 paws per CAIA; ^∗∗∗^*p* < 0.0005 and ^∗∗∗∗^*p* < 0.0001 compared to control).

Representative images of H&E staining in the radiocarpal joint are presented in Figure 4(a). Although PAR 1 mg/kg or 4 mg/kg treatment groups exhibited reduced scores for cellular infiltration, cartilage and bone degradation, and pannus formation compared to CAIA mice in the radiocarpal joint, this was not statistically significant (Figure 4(b)). Similarly, there was no significant difference in histological scores in the hind paws between all CAIA and PAR treatment groups (Figure 4(c)).

**Figure 5:**
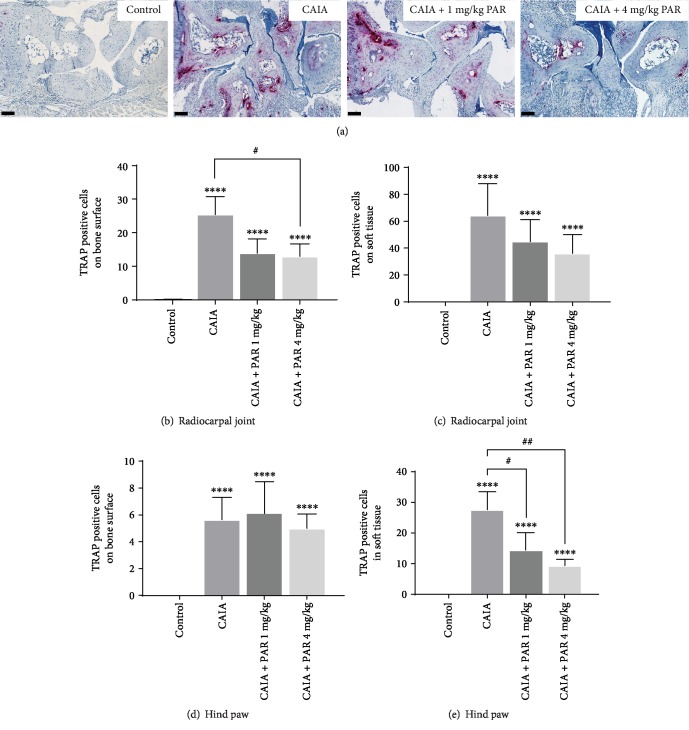
Histological assessment of osteoclast-like cells in the radiocarpal joint and hind paws: (a) representative TRAP-stained images of the radiocarpal joint (20x magnification; scale bars represent 100 *μ*m). Average values of TRAP-positive multinucleated cells on the bone surface and within the surrounding soft tissue in the radiocarpal joint (b and c, respectively) and the hind paws (d and e, respectively). Error bars represent SEM (*n* = 16 paws per control, CAIA+PAR 1 mg/kg, and CAIA+PAR 4 mg/kg, *n* = 12 paws per CAIA; ^∗∗∗∗^*p* < 0.0001 compared to control; ^#^*p* < 0.05 compared to CAIA; ^##^*p* = 0.006 compared to CAIA).

A significantly greater number of multinucleated TRAP-positive cells were observed on the bone surface and in the surrounding soft tissue of all paws in CAIA and PAR treatment groups compared to control mice (*p* < 0.0001; Figures 5(a)–5(e)). PAR 4 mg/kg-treated mice had a significantly lower number of multinucleated TRAP-positive cells on the bone surface of the radiocarpal joint compared to CAIA mice (*p* = 0.04; Figure 5(b)) and also had a reduced number of multinucleated TRAP-positive cells within the surrounding soft tissue compared to both CAIA mice and PAR 1 mg/kg-treated mice, however, this was not statistically significant (Figure 5(c)). PAR 4 mg/kg-treated mice had a lower number of multinucleated TRAP-positive cells on the bone surface of the hind paw compared to PAR 1 mg/kg-treated mice, however, this was not statistically significant (Figure 5(d)). Within the surrounding soft tissue of the hind paw, PAR 1 mg/kg- and 4 mg/kg-treated mice had significantly lower numbers of multinucleated TRAP-positive cells compared to CAIA mice (*p* = 0.025 and *p* = 0.006, respectively; Figure 5(e)). However, there was no significant difference between PAR treatment groups.

### 3.4. Histological Analysis of Glial Cells

#### 3.4.1. GFAP Expression within the Central Nervous System

**Figure 6:**
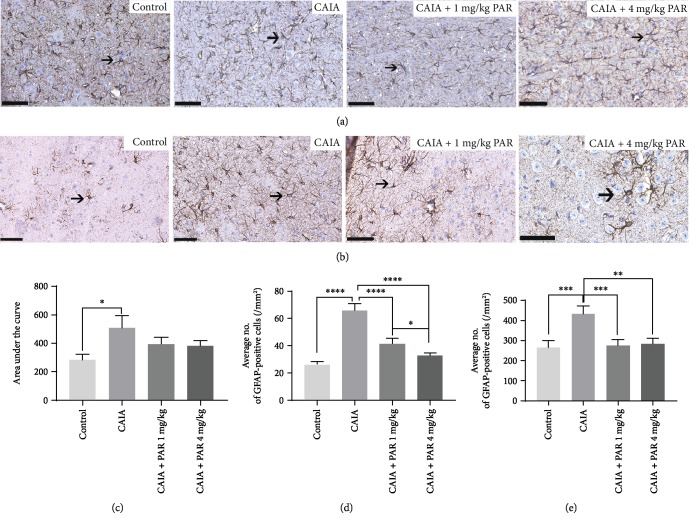
Histological assessment of GFAP-positive cells in the lumbar spinal cord region, PAG, and RVM: (a) Representative GFAP-stained image of the spinal cord (40x magnification; scale bars represent 50 *μ*m); (b) representative GFAP-stained image of the PAG (40x magnification; scale bars represent 50 *μ*m); (c) area under the curve analysis of the average number of GFAP-positive cells in serial sections within the lumbar region of the spinal cord. Average number of GFAP-positive cells in the PAG and RVM of the brain (d and e, respectively). Error bars represent SEM (*n* = 8 animals per control, CAIA+PAR 1 mg/kg, and CAIA+PAR 4 mg/kg, *n* = 6 animals per CAIA), ^∗∗∗∗^*p* < 0.00001, ^∗∗^*p* < 0.002, ^∗^*p* < 0.03.

Representative images of GFAP staining within the lumbar region of the spinal cord and PAG are represented in Figures 6(a) and 6(b), respectively. A significantly greater number of GFAP-positive cells were observed in the lumbar spinal cord, PAG, and RVM in CAIA mice compared to control mice (*p* = 0.003, *p* < 0.0001, and *p* = 0.007, respectively; Figures 6(c)–6(e)). A significant reduction in the number of GFAP-positive cells in the PAG were observed in PAR 1 mg/kg- (41.73 ± 3.90 cells/mm^2^) and 4 mg/kg- (32.72 ± 1.98 cells/mm^2^) treated mice, in comparison to CAIA mice (65.76 ± 5.26 cells/mm^2^, *p* < 0.0001; Figure 6(d)). The decrease in GFAP-positive cells in PAR 4 mg/kg-treated mice was significantly greater than that in PAR 1 mg/kg-treated mice (*p* = 0.024; Figure 6(d)). There was also a significantly lower number of GFAP-positive cells in the RVM in PAR 1 mg/kg- (275.8 ± 30.39 cells/mm^2^) and 4 mg/kg- (284.8 ± 25.69 cells/mm^2^) treated mice, compared to CAIA mice (433.0 ± 40.31 cells/mm^2^, *p* = 0.004 and *p* = 0.0013, respectively; Figure 6(e)).

#### 3.4.2. IBA1 Expression within the Central Nervous System

**Figure 7:**
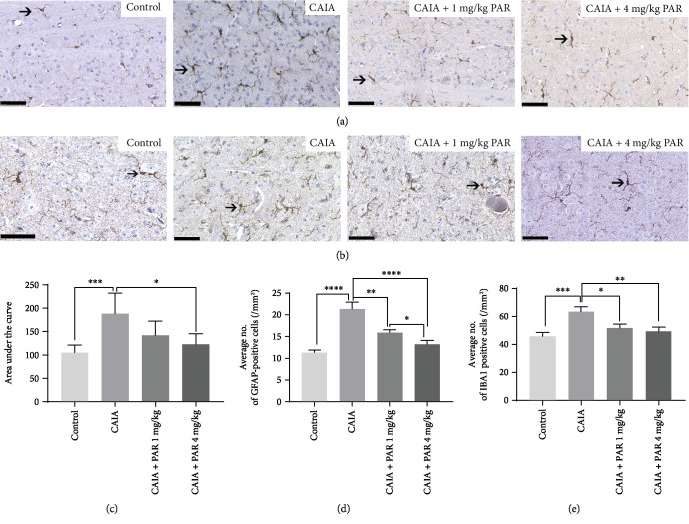
Histological assessment of IBA1-positive cells in the lumbar spinal cord region, PAG, and RVM: (a) representative IBA1-stained image of the spinal cord (40x magnification; scale bars represent 50 *μ*m); (b) representative IBA1-stained image of the PAG (40x magnification; scale bars represent 50 *μ*m); (c) area under the curve analysis of the average number of IBA1-positive cells on serial sections in the lumbar region of the spinal cord. Average number of IBA1-positive cells in the PAG and RVM of the brain (d and e, respectively). Error bars represent SEM (*n* = 8 animals per control, CAIA+PAR 1 mg/kg, and CAIA+PAR 4 mg/kg, *n* = 6 animals per CAIA), ^∗∗∗∗^*p* < 0.00001, ^∗∗^*p* < 0.002, ^∗^*p* < 0.03.

Representative images of IBA1 staining within the lumbar spinal cord and PAG are represented in Figures 7(a) and 7(b), respectively. CAIA mice had a significantly greater number of IBA1-positive cells in the lumbar spinal cord, PAG, and RVM compared to control mice (*p* < 0.0007, *p* < 0.0001, and *p* < 0.007, respectively; Figures 7(c)–7(e)). There was a significantly lower number of IBA1-positive cells in the lumbar spinal cord in PAR 4 mg/kg-treated mice (122.1 ± 8.107 cells/mm^2^) compared to CAIA mice (188.5 ± 18.02 cells/mm^2^, *p* = 0.0013; Figure 7(c)). A significant decrease in IBA1-positive cells in the PAG was observed in PAR 1 mg/kg- (15.83 ± 0.72 cells/mm^2^) and 4 mg/kg- (13.25 ± 0.86 cells/mm^2^) treated mice, compared to CAIA mice (21.28 ± 1.59 cells/mm^2^, *p* = 0.0044 and *p* < 0.0001, respectively; Figure 7(d)). A significantly lower number of IBA1-positive cells were also observed in PAR 4 mg/kg-treated mice compared to PAR 1 mg/kg-treated mice (*p* = 0.0172; Figure 7(d)). Additionally, there was a significant reduction in IBA1-positive cells in the RVM in PAR 1 mg/kg- (51.58 ± 2.80 cells/mm^2^) and 4 mg/kg- (49.33 ± 3.00 cells/mm^2^) treated mice, in comparison to CAIA mice (62.98 ± 3.90 cells/mm^2^, *p* = 0.0260 and *p* = 0.0097, respectively; Figure 7(e)).

## 4. Discussion

This study utilised the commercially available and well-established CAIA mouse model which rapidly initiates pathogenic features similar to those found in RA [23]. A milder form of the disease was induced based on previous studies within our laboratory [21, 26]. Paw inflammation was present in the mild CAIA model, as evidenced by significantly greater paw scores compared to nondiseased controls from days 5 to 10. These paw scores were greatest at day 8, consistent with the previously reported timeline of disease [23]. Paws remained inflamed at the conclusion of the model as shown by significantly greater PV in CAIA mice compared with control when assessed by *ex vivo* micro-CT. As expected, the mild CAIA model resulted in mild joint destruction and significantly lower BV in the radiocarpal joint and hind paws when compared to nondiseased controls. Glial reactivity was increased within the CAIA mice in both the spinal and supraspinal region, indicating that the model was not only able to form an inflammatory model peripherally but also has a central neuroimmune impact.

The current study found that PAR administered daily at 1 mg/kg and 4 mg/kg significantly reduced local inflammation in all paws of CAIA mice. PAR 4 mg/kg reduced local inflammation compared to CAIA mice from day 6 and was reduced even further from days 8 to 10. PAR 1 mg/kg had a lesser effect on local inflammation, as evidenced by paw scores on days 8 and 9. At day 10, levels of local inflammation were the same in both PAR-treated groups.

Micro-CT analysis of PV, as an objective measure of inflammation, was consistent with paw scores at end point. PV in CAIA mice were similar to PAR 1 mg/kg- or 4 mg/kg-treated mice, with PV being slightly lower in the radiocarpal joint in PAR-treated mice compared to CAIA mice, however, not significantly so. Although not significant, PV in the hind paws was slightly higher in PAR 1 mg/kg-treated mice. This was unexpected but could be attributed to the large variability in paw inflammation in response to the systemic administration of CAIA and PAR, as well as the lower levels of inflammation measured at endpoint in all CAIA groups.

Histological analysis of the radiocarpal joints and hind paws at endpoint also supported a reduction in local inflammation following treatment with PAR. A reduction in inflammatory cell infiltration and pannus formation was observed to be lower in PAR-treated mice compared to CAIA mice, however, no difference was observed between PAR 1 mg/kg- and 4 mg/kg-treated mice. This was unexpected, as previous studies have shown PAR 1 mg/kg to inhibit the proinflammatory effects of TNF-*α* and IL-1*β*, resulting in a reduction in inflammation and pannus formation in the synovial joints of hind paws in CIA rats [18].

PAR 1 mg/kg and 4 mg/kg treatment did not have an effect on BV in the front and hind paws of mice, whereas TRAP-positive multinucleated cells were reduced on the bone surface of the radiocarpal joint and hind paws in PAR 1 mg/kg-treated mice, with PAR 4 mg/kg-treated mice showing a greater reduction. Although the micro-CT and histology findings do not support one another, they are both consistent with a previous study, which showed PAR treatment to have a significant effect limited to the bone surface, on reducing bone surface resorption induced by polyethylene particles in a murine calvarial model of peri-implant osteolysis, but with no effect on overall BV [19]. Similarly, within the current study, it is possible that the suggestive decrease in bone resorption indicated by the decrease in TRAP-positive cells (on the bone surface) as seen by microscopic analysis could be partially compensated by the large quantity of bone (volume) analysed by micro-CT within the radiocarpal joint and hind paws. Further quantification of BV of the smaller individual carpal and tarsal bones in the front and hind paws, respectively, may be needed to show a reduction in bone loss quantifiable volumetrically in terms of BV following PAR treatment in CAIA mice.

The current study found PAR 1 mg/kg and 4 mg/kg reduced local paw inflammation, but with no change in overall BV in either front or hind paws. This is consistent with a previous study reporting a reduction in inflammation with little effect on overall bone loss, by 1 mg/kg PAR, in a CIA rat model [18]. This could be attributed to the low dose of PAR used in both treatment groups, as in the complex CAIA model, low-dose PAR may be unable to inhibit both local inflammation and the subsequent effect inflammation has on stimulating bone destruction.

PAR treatment did not reduce mechanical hypersensitivity in CAIA mice, as evidenced by inconsistent paw withdrawal thresholds. Paw withdrawal thresholds in CAIA mice remained consistent with nondiseased control thresholds from days 2-8, despite the presence of mild paw inflammation. This is not consistent with a previous study, which displayed robust mechanical hypersensitivity concomitant with the onset of joint inflammation in a moderate CAIA model in BALB/c mice [8]. Within that study, paw withdrawal thresholds, assessed using von Frey, did not return to baseline four weeks following the reduction of joint inflammation [8]. Of note, in the current study, remission stages were not investigated and the difference observed between studies may be due to the occurrence of endogenous analgesia. Previous pain models in mice have examined the effect of endogenous analgesia where mechanical hypersensitivity assessed via von Frey reached baseline withdrawal threshold scores; however, this was reversed, upon administration of naloxone, an opioid receptor antagonist [33]. This suggests that latent pain sensitisation may also occur in inflammatory pain models, such as CAIA. Future studies should incorporate automated gait analysis such as the catwalk assessment [34] when assessing pain-like behaviour in animal models of inflammatory arthritis as they examine limb guarding, a common clinical symptom in joint and neuropathic pain [35]. Additionally, as a spontaneous pain assessment, gait analysis by the catwalk could examine more complex decision-making involved in pain more accurately [35].

Mechanical hypersensitivity was hypothesised to be reduced in PAR-treated mice due to the reduction in glial cells observed, as microglia and astrocytes are associated with neuropathic pain [36, 37]. Findings from Lampa et al. suggest that nociception from an inflammatory experimental arthritis mouse model was due to changes in both the peripheral and central nervous system [38]. We propose that peripheral inflammation promoted central adaption observed via the increase in glial reactivity within our CAIA model. Kapitzke et al. 2011 reviewed multiple studies suggesting that opioid receptors were activated in inflammatory animal models [39]. Therefore, our increase in glial cells may not have aligned with a change in mechanical hypersensitivity due to peripheral analgesic responses resulting from changes in peripheral immune effector cells. Our results were suggestive of PAR acting as a neuroimmune mediator, although this was not assessed directly.

PAR has previously been shown to be effective at crossing the blood-brain barrier [40, 41]; however, few studies investigating pain and inflammation have examined glial reactivity in both the PAG and RVM. Our findings demonstrate that spinal and brain glial cell reactivity was reduced in PAR-treated mice. GFAP- and IBA1-positive cells were significantly reduced in PAR 1 mg/kg- and 4 mg/kg-treated mice compared to CAIA mice in the PAG and RVM. However, in the lumbar spinal cord, only a significant reduction of IBA1-positive cells was observed in PAR 4 mg/kg-treated mice. These findings suggest that PAR 1 mg/kg and 4 mg/kg was most effective at reducing glial reactivity within the supraspinal regions of interest, rather than the lumbar spinal cord in CAIA mice.

Sex-specific differences in both pain perception and glial reactivity have been observed within CD1 neuropathic mouse models. Vacca et al. observed that within the lumbar L4-L5 spinal cord, female mice had an increased reactivity in astrocytic and microglial cells compared to male mice [42]. Additionally, Vacca et al. measured mechanical hypersensitivity with results suggesting that female mice took longer to return to baseline than male mice [42]. Doyle et al. found that within the PAG, female Sprague Dawley rats had an increased level of microglia reactivity compared to males [43]. In future studies, the inclusion of male CAIA mice would be informative to investigate if sex-specific differences are present in the CAIA model.

The conflicting findings regarding bone loss and inflammation when compared to past studies [18, 19] may also be a result of the methodology employed to induce CAIA. Although the CAIA model has a high penetrance in BALB/c mice, the joints are randomly affected due to the systemic administration [23]. Thus, the large mouse variability in response to both disease and treatment may explain the results in the current study. There may also be variability in the absorption and availability of active metabolites of PAR within the circulation which may have also impacted the results. As PAR was not assessed in healthy mice, we are unable to identify the effect of PAR on the central nervous system in the absence of peripheral inflammation. Additionally, mechanical hypersensitivity should be assessed prior to the onset of symptoms and following remission stage as it is more clinically relevant. To achieve a more effective result in the complex *in vivo* environment, the use of an animal model that targets specific joints through the intra-articular injection of the antigen, as well as the intra-articular administration of PAR, may provide a better strategy for future studies.

## 5. Conclusion

This study demonstrated that PAR, at 1 mg/kg or 4 mg/kg, suppressed local inflammation and uniquely demonstrates the effects of peripheral inflammation on both the spinal and supraspinal nociceptive processing pathways within a mild CAIA mouse model. Further investigations involving new strategies to investigate bone and joint destruction with glial reactivity complemented by analysis of pain-like behaviour in the CAIA mouse model are warranted to clarify the role of potential therapeutics on RA specific pain, inflammation, and bone loss.

## Data Availability

The data used to support the findings of this study are available from the corresponding author upon request.

## Abbreviations

AUC: Area under the curve
BV: Bone volume
CAIA: Collagen antibody-induced arthritis
CIA: Collagen-induced arthritis
CCI: Constriction injury to the sciatic nerve
EDTA: Ethylene diaminetetraacetic acid
GFAP: Glial fibrillary acidic protein
H&E: Haematoxylin and eosin
IL-1*β*: Interleukin-1 beta
IL-6: Interleukin-6
IBA1: Ionised calcium-binding adaptor molecule 1
LPS: Lipopolysaccharide
Micro-CT: Microcomputed tomography
NF-*κ*B: Nuclear factor-kappa B
PAR: Parthenolide
PAG: Periaqueductal grey
PBS: Phosphate-buffered saline
PV: Paw volume
RA: Rheumatoid arthritis
RANK: Receptor activator of nuclear factor kappa B
RANKL: Receptor activator of nuclear factor kappa B ligand
RVM: Rostral ventromedulla
SEM: Standard error of the mean
TNF-*α*: Tumour necrosis factor alpha
TRAP: Tartrate-resistant acid phosphatase
VOI: Volume of interest.
